# Risk factors for fatality in jump racing Thoroughbreds in Great Britain (2010–2023)

**DOI:** 10.1111/evj.14450

**Published:** 2024-12-12

**Authors:** Sarah E. Allen, Sally Taylor, James Given, Kristien L. Verheyen

**Affiliations:** ^1^ Department of Pathobiology and Population Sciences Royal Veterinary College Hatfield Hertfordshire UK; ^2^ British Horseracing Authority London UK

**Keywords:** fatality, horse, racing, risk mitigation, thoroughbred, welfare

## Abstract

**Background:**

The British horseracing industry is committed to reducing equine fatalities in jump racing. Race‐related fatalities are a major welfare concern and threaten the sport's social licence to operate.

**Objectives:**

To describe the risk of, and determine risk factors for, fatality in British jump racing.

**Study design:**

Retrospective cohort.

**Methods:**

Analyses included all starts made in British jump races between January 2010 and April 2023. Available information for each horse, race, racecourse, trainer and jockey was collated and combined with details of all fatalities recorded by official veterinary officers in a central database. A fatality was defined as any post‐start veterinary event that resulted in the sudden death or euthanasia of a horse within 48 h of racing. Risk factors (*n* = 101) were evaluated using mixed‐effects logistic regression. Data for steeplechase and hurdle starts were analysed separately.

**Results:**

The overall fatality rate was 5.9 per 1000 steeplechase starts (*n* = 836/141 922; 95% CI 5.5–6.3) and 4.5 per 1000 hurdle starts (*n* = 1096/242 486; 95% CI 4.3–4.8). In both race types, fallers (steeplechase: OR 28.7, 95% CI 23.0–35.8; hurdle: OR 41.4, 95% CI 32.9–52.0) and older horses (steeplechase: OR 1.1 per extra year, 95% CI 1.1–1.2; hurdle: OR 1.2 per extra year, 95% CI 1.1–1.2) had higher odds of fatality. In steeplechase racing, starts made in summer (OR 1.2, 95% CI 1.0–1.5) and by non‐GB trained horses (OR 2.0, 95% CI 1.4–3.0) experienced higher fatality odds. In hurdling, maiden races (OR 1.3, 95% CI 1.0–1.6) were at higher odds of fatality. In both race types, softer going decreased the odds of fatality. Approximately half of the unexplained variation in fatality odds was attributable to horse.

**Main limitations:**

Analysis was restricted to routinely recorded race‐day factors and performance history.

**Conclusions:**

Reducing the risk of falling and racing on softer ground could substantially decrease fatalities in jump racing.

## 1 INTRODUCTION

Race‐related fatality is a major concern for the horseracing industry, with the sport's regulators committed to improving horse welfare and jockey safety by reducing, in as far as possible, any avoidable risk of equine fatality at the racecourse. By taking a proactive approach in addressing this welfare concern, the industry also strives to maintain the sport's social licence to operate, which is currently under threat due to growing public concern for racehorse welfare and the use of horses in sport more generally.^1^, ^2^, ^3^

In Great Britain (GB), approximately 35% of races are held over jumps.^4^ Jump racing is divided into three types: steeplechasing where horses compete over larger fences, hurdling where horses race over smaller, more flexible hurdles, and National Hunt flat racing where previously unraced jump racehorses race on the hurdle course after the hurdles have been removed. The risk of fatality and injury is inherently greater when racing over jumps compared to on the flat.^5^, ^6^, ^7^, ^8^, ^9^, ^10^ The most recently published study of fatality in British jump racing, using data from 2000 to 2009, estimated the incidence of fatality at 6.2 deaths per 1000 steeplechase starts and 4.6 deaths per 1000 hurdle starts.^8^ These figures are comparable to available fatality rates for jump racing in New Zealand^9^ and Australia.^10^ For comparison, the incidence of fatality in British flat racing, between 2000 and 2013, was 0.8 deaths per 1000 flat starts.^11^ Across all studies, fatalities are most commonly the result of catastrophic musculoskeletal injury.^8^, ^9^, ^12^, ^13^

Identifying risk factors for race‐related fatality is an important step in addressing this welfare concern, as it will allow the industry to implement changes that will effectively mitigate risk and enhance safety. Previous studies investigating jump racing fatalities have identified several risk factors including increasing age, longer race distances and firmer going.^6^, ^8^, ^14^, ^15^, ^16^, ^17^, ^18^, ^19^ Prior racing intensity and season have also been shown to affect fatality risk.^6^, ^19^

In the last 10 years, there have been no studies that have estimated the incidence of, or evaluated risk factors for, equine fatality in British jump racing. Therefore, the objectives of this work were to provide up‐to‐date estimates of race‐related fatality incidence and identify current risk factors for fatality in jump racing Thoroughbreds in GB. Knowledge of the current factors influencing risk is essential for the design and implementation of effective strategies that mitigate the risk of fatality and advance racehorse and jockey safety.

## 2 MATERIALS AND METHODS

The study population included all steeplechase and hurdle race starts made by Thoroughbred racehorses on British racecourses between 1 January 2010 and 30 April 2023. Routinely recorded data on these starts were extracted from Weatherbys' racing database,^20^ including information on horse demographics and performance history, race conditions, course information and trainer and jockey race experience. These data were combined with details of all race‐related fatalities, recorded by official veterinary officers in the British Horseracing Authority's (BHA's) purpose‐built injury database. Race‐related fatality was defined as any post‐start race‐related veterinary event that resulted in the sudden death or euthanasia (including elective euthanasia) of a horse within 48 h of racing. All causes of fatality were included. Data for steeplechase and hurdle race starts were analysed separately.

In this study, the unit of observation was a race start, that is, a horse that crossed the starting tape, and one horse could make multiple starts during the study period. Incidence of fatality was expressed as the number of deaths per 1000 starts and associated 95% confidence intervals calculated. Causes of fatality were described using counts and percentages.

A total of 101 potential risk factor variables were evaluated (57 horse‐related, 27 race‐related, 6 course‐related, 6 trainer‐related, 5 jockey‐related) (Table S1). Age‐related variables included current age (in years), age at first race start and the number of years in racing. Horse sex was categorised as male (colts, stallions and geldings) or female (fillies and mares). Initially, the firmness of the racing surface or going was categorised into six groups: firm, good‐to‐firm, good, good‐to‐soft, soft and heavy. Performance variables were calculated using data prior to the race start of interest and included the number of previous race starts, percentage wins, percentage placed and average performance score (30 points awarded for a win, 20 for a place, 10 for a completed run, 0 for a start when the horse failed to finish).^8^, ^21^ As the racing database only stores information for starts made in GB and outside GB if the race includes a GB‐trained horse, performance variables for non‐GB participants were set to missing as the available performance history may not have been complete. Course topography and ‘speed’ characteristics were reported in the database. These characteristics were based on the subjective opinion of an individual assessor with expertise in racecourse inspection. Course topography was categorised as either flat, undulating or very undulating. Course ‘speed’ was categorised as either galloping, stiff, tight, sharp or very sharp. This measure is an indicator of course shape, with galloping courses having gentle bends and very sharp courses the most acute bends. Exposure variables were initially screened using univariable logistic regression and those with a likelihood ratio test (LRT) *p* value <0.25 were carried forward for consideration in the multivariable model. Continuous variables were assessed for a linear association with the outcome and if non‐linearity was identified by either the LRT or graphical assessment, the variable was categorised based on rounded quartiles. After ranking variables to be taken forward for multivariable modelling by the Akaike information criterion (AIC) and log likelihood values, models were built using a forward selection process, starting with the variable with the lowest AIC. Variables were retained if the LRT *p* values were ≤0.05.

All variables carried forward for multivariable modelling but not included in the final models were resubmitted to check for potential confounding. Confounding was considered present if the addition of a variable altered the estimates by >10%, in which case the confounding variable was retained. Biologically plausible two‐way interactions between variables in the final model were assessed by comparing models including and excluding interaction terms using the LRT. Mixed‐effects logistic regression modelling was used throughout, including horse as a random effect to account for horses making multiple starts during the study period. Due to computational constraints, it was not possible to generate cross‐classified models featuring multiple random effects. To check for potential clustering by trainer, jockey and racecourse within the final model, the random effect of horse was replaced by an individual random effect term for each potential cluster and between‐cluster variation (sigma) and within‐cluster correlation (rho), which estimates the proportion of variances in the outcome that is attributable to the cluster variable, calculated.

Power calculations, based on the available number of race starts and observed fatality rates, indicated that both the steeplechase and hurdle analyses had at least 80% power to detect an odds ratio of 1.5 or more, at 95% confidence, if the prevalence of exposure amongst the non‐fatal starts was between 5% and 91%. Statistical analyses and power calculations were performed in Stata 14 (StataCorp LLC, College Station, Texas).

## 3 RESULTS

### 3.1 Description of fatality in jump racing

During the study period, there were 384 408 jump starts of which 1932 ended in fatality. This equates to an overall incidence of 5.0 fatalities per 1000 jump starts (95% CI 4.8–5.3). When considering the two race codes separately, the incidence of fatality was 5.9 per 1000 steeplechase starts (836/141 922; 95% CI 5.5–6.3) and 4.5 per 1000 hurdle starts (1096/242 486; 95% CI 4.3–4.8). Causes of fatality are summarised in Table 1. The most common cause of fatality was catastrophic musculoskeletal injury, accounting for 86.5% of steeplechase and 89.3% of hurdle deaths. Overall, 10.8% of fatalities were recorded as being due to a confirmed or presumptive cardiovascular incident.

**TABLE 1:** Description of all fatalities associated with jump racing in Great Britian between 1 January 2010 and 30 April 2023.

	Number of fatalities in jump starts (%^a^)	Number of fatalities in steeplechase starts (%^a^)	Number of fatalities in hurdle starts (%^a^)
Musculoskeletal	1702 (88.1)	723 (86.5)	979 (89.3)
Fracture	1291 (66.8)	586 (70.1)	705 (64.3)
Tendon or ligament injury—severe injury/breakdown	192 (9.9)	64 (7.7)	128 (11.7)
Tendon or ligament injury—lacerated/severed	173 (9.0)	49 (5.9)	124 (11.3)
Tendon or ligament injury—moderate strain	17 (0.9)	5 (0.6)	12 (1.1)
Tendon or ligament injury—dislocation/avulsion	4 (0.2)	2 (0.2)	2 (0.2)
Tendon/ligament injury—type not reported	1 (0.1)	1 (0.1)	0 (0.0)
Joint luxation/dislocation	49 (2.5)	25 (3.0)	24 (2.2)
Joint effusion	2 (0.1)	0 (0.0)	2 (0.2)
Bruise or haematoma	3 (0.2)	1 (0.1)	2 (0.2)
Musculoskeletal injury—type not reported	2 (0.1)	1 (0.1)	1 (0.1)
Cardiovascular^b^	208 (10.8)	101 (12.1)	107 (9.8)
Vascular catastrophe	201 (10.4)	98 (11.7)	103 (9.4)
Cardiac arrhythmia	2 (0.1)	1 (0.1)	1 (0.1)
Cardiovascular episode—type not reported	5 (0.3)	2 (0.2)	3 (0.3)
Other	20 (1.0)	13 (1.6)	7 (0.6)
Wound	7 (0.4)	3 (0.4)	4 (0.4)
Epistaxis	6 (0.3)	6 (0.7)	0 (0.0)
Collapse ‐ type not reported^b^	3 (0.2)	3 (0.4)	0 (0.0)
Cerebral lesion	3 (0.2)	1 (0.1)	2 (0.2)
Spinal lesion	1 (0.1)	1 (0.1)	0 (0.0)
Colic	1 (0.1)	0 (0.0)	1 (0.1)
Type not reported	12 (0.6)	6 (0.7)	6 (0.5)

^a^ Sum to more than 100% as more than one injury type could be reported for each fatality.

^b^ Post‐mortem confirmation was not performed in all cases thus some diagnoses are presumptive and based on observations made by the attending veterinarians.

### 3.2 Risk factors for fatality in steeplechase racing

The steeplechase dataset consisted of 141 922 race starts made by 19 134 horses in 19 126 races. Of these steeplechase starts, 836 resulted in fatality. A total of 2390 trainers and 1684 jockeys contributed to this dataset. Initial screening of potential risk factors resulted in 38 being taken forward to the multivariable model build. Descriptive statistics and univariable results for variables taken forward to multivariable analysis are presented in Table S2.

The final multivariable model results showing factors associated with fatality in steeplechase racing are presented in Table 2. The eight identified risk factors were horse age, horse sex, faller status in race, faller status prior to race, jump season, going, race value and country where the horse was trained.

**TABLE 2:** Results of mixed‐effects multivariable logistic regression analysis investigating risk factors for fatality in horses competing in steeplechase races in Great Britain between 1 January 2010 and 30 April 2023.

Risk factor	Starts (*n*)	Fatalities (*n*)	Fatality incidence per 1000 starts	Odds ratio	95% Confidence interval	LRT *p*‐value
						Wald *p*‐value
Age at race						**<0.001**
Per extra year	141 922	836	5.9	1.14	1.08–1.21	*<0.001*
Sex at race						**0.020**
Male	130 096	780	6.0	Reference		
Female	11 826	56	4.7	0.68	0.49–0.96	*0.026*
Horse fell in race						**<0.001**
No	136 151	464	3.4	Reference		
Yes	5771	372	64.5	28.71	23.04–35.78	*<0.001*
Horse previously fell in a race						**0.034**
No & unraced	65 866	362	5.5	Reference		
Yes	33 575	206	6.1	1.36	1.07–1.73	*0.012*
Incomplete data	42 481	268	6.3	1.12	0.91–1.39	*0.248*
Jump season						**0.049**
Core (Oct–Apr)	111 195	627	5.6	Reference		
Summer (May–Sep)	30 727	209	6.8	1.22	1.00–1.50	*0.048*
Going						**<0.001**
Firm & good to firm	7830	55	7.0	1.21	0.85–1.73	*0.286*
Good	53 827	366	6.8	1.19	0.97–1.45	*0.097*
Good to soft	33 558	200	6.0	Reference		
Soft	32 637	163	5.0	0.79	0.62–1.00	*0.046*
Heavy	14 070	52	3.7	0.59	0.42–0.84	*0.003*
Race value						**0.028**
£1150–6000	41 656	230	5.5	Reference		
£6001–8700	29 554	194	6.6	1.40	1.12–1.75	*0.003*
£8701–15 000	37 250	202	5.4	1.09	0.87–1.37	*0.436*
More than £15 000	33 462	210	6.3	1.12	0.88–1.41	*0.369*
Trainer country						**<0.001**
Great Britain	138 085	785	5.7	Reference		
Other	3837	51	13.3	2.02	1.37–2.99	*<0.001*
*Random effect*						
Sigma				1.78	1.42–2.22	
Rho				0.49	0.38–0.60	**<0.001**

*Note*: Horse ID was included as a random effect. The bold values are the *p*‐values for the likelihood ratio test (LRT). The italicised values are the *p*‐value for the Wald test.

Horses that fell during a steeplechase race had 29 times the odds of fatality compared to non‐fallers (OR 28.71, 95% CI 23.04–35.78, *p* < 0.001). After adjusting for the effects of falling in the race and the other factors in the model, steeplechase starts made by horses that had previously fallen in a race were also at higher odds of fatality compared to those that had not (OR 1.36, 95% CI 1.07–1.73, *p* = 0.03). A horse's age at the time of the race was significantly associated with the odds of fatality. For every 1‐year increase in age, the odds of fatality in steeplechase races increased by 14% (OR 1.14, 95% CI 1.08–1.20, *p* < 0.001). Compared to male horses, lower odds of fatality were observed for steeplechase starts made by female horses (OR 0.68, 95% CI 0.49–0.96, *p* = 0.02). The odds of fatality were 22% higher for steeplechase starts made during the summer jump season (May–September) compared to those in the core season (October–April) (OR 1.22, 95% CI 1.00–1.50, *p* < 0.05). After adjusting for the effect of season, steeplechase starts made on heavy and soft going had significantly lower odds of fatality compared to those made on good‐to‐soft going (*p* < 0.05). There was no statistically significant difference in the odds of fatality between steeplechase starts made on good‐to‐soft going and those made on firmer going. Steeplechase starts made by horses trained outside of GB had twice the odds of fatality compared to those made by horses trained in GB (OR 2.02, 95% CI 1.37–2.99, *p* < 0.001).

Significant between‐horse variation was observed (*p* < 0.001), with rho indicating that 49% of the unexplained variation in the odds of fatality was attributable to horse. This supported the inclusion of horse as a random effect. There was also evidence of significant variation between trainers (*p* < 0.01) and racecourses (*p* = 0.03) although the proportions of variation in fatality outcome explained by trainer and racecourse were only 2% and 1%, respectively.

### 3.3 Risk factors for fatality in hurdle racing

The hurdle dataset consisted of 242 486 race starts made by 37 499 horses across 26 109 races. Of these hurdle starts, 1096 resulted in fatality. A total of 1323 trainers and 1343 jockeys contributed to this dataset. Initial screening of potential risk factors resulted in 42 being taken forward to the multivariable model build. Descriptive statistics and univariable results for variables taken forward to multivariable analysis are presented in Table S3.

The final multivariable model results showing factors associated with fatality in hurdle racing are presented in Table 3. The six identified risk factors included horse age, faller status, going, maiden races, course speed description and the trainer's career average win rate. As in the steeplechase model, faller status also had the strongest association with fatality odds in hurdle racing. Horses that fell in a hurdle race had 41 times the odds of fatality compared to non‐fallers (95% CI 32.89–51.99, *p* < 0.001). Also as observed in the steeplechase model, older horses were at greater odds of fatality, with every 1‐year increase in horse age, increasing the odds of fatality in hurdle racing by 18% (OR 1.18, 95% CI 1.13–1.24, *p* < 0.001). Compared to hurdle starts ran on good‐to‐soft going, those made on firmer going had greater odds of fatality (*p* < 0.01) and those made on softer going had lower odds of fatality (*p* < 0.01). For starts made in hurdle races restricted to maiden horses, there was a 28% increase in the odds of fatality compared to non‐restricted races (OR 1.28, 95% CI 1.05–1.56, *p* = 0.02). The type of racetrack influenced the odds of fatality, with a 17% increase in the odds of fatality observed for hurdle starts made on ‘stiff or tight’ tracks compared to ‘galloping’ ones (OR 1.17, 95% CI 1.02–1.34, *p* = 0.02). The odds of fatality were 26% greater for hurdle starts made for GB trainers with an average career win rate greater than 15% (the top 25% of trainers) compared to those made for GB trainers with an average career win rate below 15% (OR 1.26, 95% CI 1.06–1.49, *p* < 0.01). Since complete performance information was not available for non‐GB trainers, this group was labelled as an ‘incomplete data’ group for the purpose of analysis; this group was not significantly different from the baseline category in terms of fatality odds.

**TABLE 3:** Results of mixed‐effects multivariable logistic regression analysis investigating risk factors for fatality in horses competing in hurdle races in Great Britain between 1 January 2010 and 30 April 2023.

Risk factor	Starts (*n*)	Fatalities (*n*)	Fatality incidence per 1000 starts	Odds ratio	95% Confidence interval	LRT *p*‐value
						*Wald p‐value*
Age at race						**<0.001**
Per extra year	242 486	1096	4.5	1.18	1.13–1.24	*<0.001*
Horse fell in race						**<0.001**
No	237 825	734	3.1	Reference		
Yes	4661	362	77.7	41.35	32.89–51.99	*<0.001*
Going						**<0.001**
Firm & good to firm	14 287	94	6.6	1.58	1.20–2.06	*0.001*
Good	93 167	517	5.5	1.28	1.08–1.52	*0.004*
Good to soft	56 615	251	4.4	Reference		
Soft	55 642	182	3.3	0.72	0.58–0.88	*0.002*
Heavy	22 775	52	2.3	0.50	0.36–0.69	*<0.001*
Race restricted to maidens						**0.022**
No	212 660	937	4.4	Reference		
Yes	29 826	159	5.3	1.28	1.05–1.56	*0.015*
Course speed description						**0.023**
Galloping	110 395	456	4.1	Reference		
Stiff, tight, sharp or very sharp	132 091	640	4.8	1.17	1.02–1.34	*0.024*
Trainer career average win rate (%)						**0.025**
≤15.0%	181 917	787	4.3	Reference		
>15% (top quartile)	54 269	269	5.0	1.26	1.06–1.49	*0.008*
Incomplete data	6260	40	6.3	1.24	0.84–1.83	*0.273*
*Random effect*						
Sigma				1.85	1.53–2.24	
Rho				0.51	0.42–0.60	**<0.001**

*Note*: Horse ID was included as a random effect. The bold values are the *p*‐values for the likelihood ratio test (LRT). The italicised values are the *p*‐value for the Wald test.

Again, significant between‐horse variation was observed (*p* < 0.001), with rho indicating that 51% of the unexplained variation in the odds of fatality was attributable to horse. There was also evidence of significant variation between trainers (*p* < 0.001), however, the proportion of unexplained variation in the odds of fatality attributable to trainer was only 3%.

## 4 DISCUSSION

This study used routinely available data to inform on the current risks of, and risk factors for, fatality in jump racing Thoroughbreds in GB. The reported rates and identified risk factors are broadly consistent with previous investigations of fatality in British jump racing. Results from this study can be used to support the development of appropriate risk‐mitigating strategies, evaluate the potential impact of future interventions and as a benchmark for the continued monitoring of race‐related fatality.

The reported fatality rates are similar to those previously estimated for British jump racing^8^, ^18^ and more recently for jump racing in New Zealand^9^ and Australia.^10^ Across all studies, the incidences of fatality ranged for 3.3–5.7 per 1000 jump (combined steeplechase and hurdle) starts, with the lowest estimate describing the rate for the 2022 and 2023 Australian jump season.^10^ Although some of the variation between racing jurisdictions may be explained by differences in the underlying horse populations and course design, care is also needed when making comparison to countries, like Australia, hosting relatively few jump races, due to the greater level of uncertainty surrounding their point estimates. While comparison between studies of British jump racing is suggestive of a downward trend in fatality rate over time, the magnitude of difference is not statistically significant. The lack of a significant reduction in fatality rates in British jump racing, over the last 20 years, highlights the importance of the assessment and identification of risk factors to direct the changes needed to improve horse and jockey safety.

In agreement with past works, fatality was most commonly attributed to catastrophic musculoskeletal injury.^8^, ^9^, ^12^, ^13^ The proportion of fatalities attributable to confirmed or presumptive cardiovascular causes, in the current study, was similar to that previously reported by Reardon et al. (2013) in British Jump racing between 2000 and 2009 (13.0%; 95% CI 11.3–14.9)^8^; however, greater than that described by Bennet and Parkin (2022) for flat racing in the United States between 2009 and 2014 (7.4%; 95% CI 6.8–8.1).^22^ Post‐mortem examination was not performed in all cases thus the causes of death were as reported by the attending veterinary officers and were often based on clinical examination only.

Three factors were common to both the steeplechase and hurdle fatality models: faller status, horse age and going. Faller status had the strongest association with fatality odds, with fallers being 29 and 41 times more likely to die compared to non‐fallers in steeplechase and hurdle races, respectively. Although equine fatalities in jump racing are often associated with falling, faller status has rarely been examined as a risk factor for fatality. This may be because of the difficulty in determining whether the fall was the result of, or resulted in, fatality. Quantifying this association does, however, demonstrate the benefit of also understanding the risk factors for horse falls, as by reducing the risk of fall, there is also likely to be a reduction in fatality.

Increasing horse age was associated with increased fatality odds in both steeplechase and hurdle racing. This is consistent with previous investigations of all‐cause fatality^6^, ^8^, ^18^ and catastrophic musculoskeletal injury.^23^ A longer life likely provides greater opportunity for the accumulation of bone microdamage and structural deterioration of tendons, thereby predisposing older horses to catastrophic musculoskeletal injury. Another potential explanation is that treatment for severe injury may be less likely sought for older horses, however, in the current study, there was still a similar effect of age after exclusion of elective euthanasia cases (data not shown). Older horses have also been shown to be at increased risk of sudden death,^22^, ^24^ with it again hypothesised that either structural changes to the cardiovascular system in response to training or microdamage accumulation may predispose these older animals to sudden death.

The firmness of the racing surface was associated with fatality in both models, with decreased odds of death observed for starts made on softer ground. Similar associations between going and race‐related fatality have been observed in studies of risk across all race types,^6^, ^18^ in flat racing^11^, ^25^ and in jump racing,^8^ whereby firmer going was found to increase the odds of fatality. Conversely, an investigation by Lyle et al. (2012) indicated that starts made on soft and heavy going had greater odds of sudden death compared to those made on good ground.^24^ The observed decreased fatality risk on softer going may be attributable to slower race speeds, however, the winner's speed was not found to affect the outcome in the current work.

Additional factors associated with increased odds of fatality in steeplechase racing included starts made in the summer jump season (May–September), by male horses and by horses trained outside GB. With the firmness of the racing surface controlled for, within the model, the effect of jump season is an interesting one as it is independent of going. One thought is that higher temperatures during the summer months may contribute to horse fatigue and injury. Other potential explanations include altered hoof‐track interactions resulting from increased artificial watering in the warmer, drier summer months to maintain the optimal going, and differences in the experience and ability of horses competing in summer jumping compared to the core jump season. To test these hypotheses, future risk factor analyses should look to incorporate weather information and data relating to artificial watering and ground maintenance. It is also unclear why horses trained outside GB should be at greater risk of fatality although this could perhaps be explained by differences in training practices and race experience. In addition, Pinchbeck et al. (2004) determined that longer journey times from training yard to racecourse were associated with a greater risk of fall in steeplechase and hurdle racing.^26^ With faller status strongly associated with fatality odds, this prior observation may partly explain the greater odds of fatality seen amongst non‐GB trained horses in the current study, who have likely had a longer journey to the racecourse compared to their GB trained counterparts. Alongside specific evaluation of training and past performances, enhanced pre‐race veterinary inspection of horses trained outside GB may help elucidate the reasons for this observation and ensure their suitability to race.

The additional factors associated with increased fatality odds in hurdling were starts made in maiden races, on stiff or tight courses and for trainers in the top 25% when ranked by average win rate. Maiden hurdle races are restricted to horses that have not previously won a hurdle race. The fact that maiden races are, therefore, likely contested by horses with less race experience and perhaps of lesser ability may explain the increased fatality odds observed under these race conditions. Conflicting with what was found in the current study, Reardon (2013) found that starts made in maiden or novice races were at lower odds of fatality compared to races not restricted to maidens or novices.^8^ In agreement with Reardon (2013), this study also found that starts made for the most successful trainers had the greatest likelihood of fatality.^8^ Further work is needed to understand whether this can be attributable to differences in training practices and/or the quality and competitiveness of horses at different training yards.

The main limitations of this work relate to the completeness and breadth of information in the racing databases. The accessed databases collate information about all races held on British racecourses but only details of races outside GB if featuring a GB‐trained horse. As such, it was not possible to evaluate the effects of experience and performance history for non‐GB‐trained horses or non‐GB trainers. Greater collaboration between racing jurisdictions including the sharing of performance histories for horses competing in GB would potentially provide useful insight into why horses trained outside of GB are at greater risk of fatality when racing in GB. Being restricted to data available within the racing databases, analyses were largely restricted to evaluation of race‐day conditions and performance history. With much of the unexplained variation in the odds of fatality attributed to horse, future work should look to incorporate other horse‐related factors, not explored in the current study, such as training regime, injury history and medication records.

Assessment of a composite outcome may also be considered a limitation, as it assumes that risk factors are the same for all causes of fatality. The significant effects of some risk factors associated with specific causes may, therefore, have been diluted if the same factor was not also associated with other causes or had an opposing effect on a different cause. That said, previous studies of fatal injury and sudden death have identified common risk factors,^8^, ^11^, ^15^, ^16^, ^17^, ^18^, ^19^, ^22^, ^24^, ^25^, ^27^ suggesting that a more generic approach is likely to be informative for reducing race‐related fatality.

This study has identified several risk factors for equine fatality in British jump racing including horse age, faller status and going. Although many of the identified risk factors only have a marginal effect or apply to a small number of starts, if a multi‐faceted approach is taken to risk mitigation, the aggregation of marginal gains could lead to a substantial decrease in the overall risk of race‐related fatality. Efforts to minimise avoidable risk must be sustained and prioritised by the racing industry.

## FUNDING INFORMATION

This study was supported by The Racing Foundation through the Horse Welfare Board.

## CONFLICT OF INTEREST STATEMENT

The authors declare no conflicts of interest.

## AUTHOR CONTRIBUTIONS

**Sarah E. Allen:** Conceptualization; data curation; formal analysis; methodology; project administration; validation; visualization; writing – original draft; writing – review and editing. **Sally Taylor:** Conceptualization; funding acquisition; investigation; project administration; writing – review and editing. **James Given:** Conceptualization; funding acquisition; investigation; project administration; writing – review and editing. **Kristien Verheyen:** Conceptualization; formal analysis; funding acquisition; methodology; project administration; supervision; resources; writing – review and editing.

## DATA INTEGRITY STATEMENT

Sarah E. Allen had full access to all data in the study and takes responsibility for the integrity of the data and the accuracy of the data analysis.

## ETHICAL ANIMAL RESEARCH

Ethical approval was obtained from the Royal Veterinary College's Clinical Research and Ethical Review Board (URN 2023 2182‐2).

## INFORMED CONSENT

Representatives of the British Horseracing Authority gave consent for the use of their data in this research.

## Supporting information


**Table S1:** Description of assessed variables.

**Table S2:** Descriptive statistics and results of univariable logistic regression for fatality in steeplechase racing.

**Table S3:** Descriptive statistics and results of univariable logistic regression for fatality in hurdle racing.

## Data Availability

The data that support the findings of this study are available from the British Horseracing Authority. Restrictions apply to the availability of these data, which were used under licence for this study.
