# Supplementary material for: Risk factors for fatality in jump racing Thoroughbreds in Great Britain (2010–2023)
Source: Equine Vet J. 2024 Dec 12;57(4):870–7. doi: 10.1111/evj.14450 (PMC12135743; doi:10.1111/evj.14450)
Supplement: Supplementary file 1 — Table S1: Description of assessed variables. [file EVJ-57-870-s002.pdf]

Table S1: Variables assessed.

VARIABLE NAME	DEFINITION
**Horse factors**	
Country of birth	Horse's country of birth
Age horse entered training	Age in years when entered training
Age at first start	Age in years at first race start
First race start	Horse's first race start (y/n)
First jump start	Horse's first jump start (y/n)
First chase/hurdle start	Horse's first chase/hurdle start (y/n)
First race type	Horse's first race type e.g. flat, NHF, hurdle, chase
Years in racing	Years since a horse's first start
Horse career starts (all race types)	Total number of starts made by horse in all race types
Horse career starts (jump)	Total number of starts made by horse in jump races
Horse career starts (chase/hurdle)	Total number of starts made by horse in chase/hurdle races
Horse career % flat	Percentage of career starts made on flat (ordinary and NHF)
Horse career win rate (all race types)	% starts won in all race types
Horse career place rate (all race types)	% starts placed in all race types
Horse career average score (all race types)	average score in all race types
Horse career noncomplete rate (all race types)	% starts horse did not complete in all race types
Horse career fall rate (all race types)	% starts horse fell in all race types
Horse career distance (all race types)	Total distance ran by horse in all race types
Horse starts in last 7 days	Number of starts made by horse in last 7 days (any race type)
Horse starts in last 14 days	Number of starts made by horse in last 14 days (any race type)
Horse starts in last 30 days	Number of starts made by horse in last 30 days (any race type)
Horse starts in last 60 days	Number of starts made by horse in last 60 days (any race type)
Horse starts in last 90 days	Number of starts made by horse in last 90 days (any race type)
Horse starts in last 180 days	Number of starts made by horse in last 180 days (any race type)
Horse starts in last 365 days	Number of starts made by horse in last 365 days (any race type)
Days since last run	Number of days since horse last raced
Age at start	Age in years at race start
Sex at start	Sex at race start
Favourite	Horse starting as favourite (y/n)
BHA rating	Official BHA rating at time of race
Top Band Handicap	At top of handicap band
Unrated	Horse without official BHA rating at time of race (y/n)
Weight carried	Weight (lb) carried in race
Horse penalty	Horse running with a penalty (y/n)
Horse penalty (lb)	Horse penalty expressed in pounds
Jockey claim	Jockey claimed weight allowance (y/n)
Jockey claim (lb)	Jockey claim allowance expressed in pounds
Racing out of handicap	Horse racing out of handicap (y/n)
Racing out of handicap (lb)	Number of pounds horse racing out of handicap
Eye covering	Horse wore a type of eye covering i.e. blinkers, cheekpieces, eye covers, shields or visor (y/n)
Hood	Horse wore a hood (y/n)

Horse factors	
Tongue strap	Horse worse a tongue strap (y/n)
Change in race type	Change in race type since horse last race (y/n)
Change in race class	Change in race class e.g. higher - same - lower
Change in weight carried	Change in weight carried e.g. more - same - less
Change in race distance	Change in race distance e.g. further - same - shorter
Change in going category	Change in going e.g. - firmer - same - softer
Change of jockey	Different jockey since last race (y/n)
Change of trainer	Different trainer since last race (y/n)
Horse non-complete in last race	Horse non-complete in last race (y/n)
Horse fell in race	Horse fell in race (y/n)
Horse fell in last race	Horse fell in last race (y/n)
Previous LTI	Horse had previous race-related LTI (y/n)
Days since first LTI	Days since first LTI
Days since last LTI	Days since last LTI
Number of LTIs in last 180 days	Number of LTIs in last 180 days
Number of LTIs in last 365 days	Number of LTIs in last 365 days
**Race factors**	
Race year	Year of race
Race month	Month of race
Season	Meteorological season i.e. spring, summer, autumn, winter
Jump season	Core (Oct-Apr) or summer (May-Sep) jump season
Race time	Time of race e.g. morning, afternoon, evening
Field size	Number of runners
No. of places	Number of places awarded prize money
Race distance	Distance of race (metres)
Going	Going
Direction of race	Direction of race e.g. left- or right-handed, figure 8
Race class	Class of race
Purse value	Value of race purse
Hurdle type	Type of hurdle i.e. birch, brush or padded
Pattern race	Pattern race (y/n)
Pattern grade	Pattern grade
Listed race	Listed race (y/n)
Handicap race	Weights carried allocated by handicapper (y/n)
Amateur race	Restricted to amateur jockeys (y/n)
Conditional race	Restricted to conditional jockeys (y/n)
Maiden race	Restricted to maiden horses (y/n)
Novice race	Restricted to novice horses (y/n)
Hunter race	Restricted to amateur jockeys and horses with hunting certificate (y/n)
Auction race	Restricted to horses brought from public auction below a price limit (y/n)
Seller race	Race in which winner offered for auction post-race (y/n)
Claimer race	Race in which every horse can be bought post-race (y/n)
Fillies race	Restricted to fillies (y/n)
Winning Speed	Speed of winner (m/s)
**Course factors**	
Course topography	Course topography i.e. flat or undulating
Course speed description	Course speed i.e. galloping, tight or stiff

Number of runners within last 30 days	Number of runners at course within last 30 days
Number of runners within last 90 days	Number of runners at course within last 90 days
Number of runners within last 180 days	Number of runners at course within last 180 days
Number of runners within last 365 days	Number of runners at course within last 365 days
**Trainer factors**	
Trainer license type	Trainer license type e.g. combined, jump, permit
Trainer country	Country trainer based in
Trainer years raced (all race types)	Years since trainer's first run
Trainer career runs (all race types)	Total number of starts made by trainer in all race types
Trainer career win rate (all race types)	Percentage of starts won by trainer in all race types
Trainer career place rate (all race types)	Percentage of starts placed by trainer in all race types
**Jockey factors**	
Jockey license type	Jockey license type e.g. amateur, conditional, professional
Jockey years raced (all race types)	Years since jockey's first ride
Jockey career rides (all race types)	Total number of starts made by jockey in all race types
Jockey career win rate (all race types)	Percentage of starts won by jockey in all race types
Jockey career place rate (all race types)	Percentage of starts placed by jockey in all race types
