# Supplementary material for: Risk factors for fatality in jump racing Thoroughbreds in Great Britain (2010–2023)
Source: Equine Vet J. 2024 Dec 12;57(4):870–7. doi: 10.1111/evj.14450 (PMC12135743; doi:10.1111/evj.14450)
Supplement: Supplementary file 2 — Table S2: Descriptive statistics and results of univariable logistic regression for fatality in steeplechase racing. [file EVJ-57-870-s003.pdf]

Equine Veterinary Journal BEVA

Table S2: Descriptive statistics and results of univariable logistic regression for fatality in steeplechase racing

	DESCRIPTIVE STATISTICS					UNIVARIABLE (RANDOM EFFECT - HORSE)						
	non-fatal	fatal	total	% fatal		OR	SE	Z	P	95% CI		LRT
**YEARS IN RACING**												
	median	IQR	range									
	4	3 to 6	1 to 14									
1 to 3	33510	174	33684	0.52		**ref**						0.001
4	21201	128	21329	0.60		1.359	0.177	2.350	0.019	1.052	1.756	
5 to 6	29038	166	29204	0.57		1.438	0.189	2.770	0.006	1.112	1.860	
more than 6	15124	100	15224	0.66		1.977	0.323	4.170	0.000	1.435	2.724	
horse data incomplete	42213	268	42481	0.63		1.503	0.182	3.360	0.001	1.185	1.906	
					sigma	1.856	0.153			1.579	2.181	
					rho	0.511	0.041			0.431	0.591	0.000
**HORSE CHASE STARTS**												
	median	IQR	range									
	7	3 to 13	1 to 97									
1 to 3	61346	381	61727	0.62		**ref**						0.178
4 to 7	17655	108	17763	0.61		1.170	0.148	1.240	0.215	0.913	1.499	
8 to 13	11752	48	11800	0.41		0.852	0.152	-0.900	0.367	0.601	1.208	
14 to 97	8120	31	8151	0.38		0.858	0.192	-0.690	0.493	0.554	1.329	
horse data incomplete	42213	268	42481	0.63		1.140	0.108	1.380	0.166	0.947	1.373	
					sigma	1.505	0.196			1.166	1.942	
					rho	0.408	0.063			0.292	0.534	0.000
**HORSE CAREER WIN RATE**												
	median	IQR	range									
	14.3	6.9 to 21.7	0 to 100									
0 to 6.9	24540	127	24667	0.51		**ref**						0.111
7.0 to 14.2	27151	147	27298	0.54		1.188	0.161	1.270	0.203	0.911	1.550	
14.3 to 21.7	21979	139	22118	0.63		1.447	0.204	2.620	0.009	1.097	1.909	
21.8 to 100	24429	150	24579	0.61		1.256	0.172	1.660	0.097	0.960	1.643	
unraced	774	5	779	0.64		1.113	0.534	0.220	0.823	0.435	2.852	
horse data incomplete	42213	268	42481	0.63		1.371	0.171	2.530	0.011	1.074	1.752	
					sigma	1.628	0.152			1.356	1.955	
					rho	0.446	0.046			0.359	0.537	0.000

**HORSE CAREER PLACE RATE**												
	median	IQR	range									
	37.5	27.8 to 47.1	0 to 100									
0 to 27.8	24649	138	24787	0.56		**ref**						0.175
27.9 to 37.5	25592	159	25751	0.62		1.217	0.156	1.530	0.125	0.947	1.564	
37.6 to 47.1	23542	142	23684	0.60		1.200	0.161	1.360	0.175	0.922	1.562	
47.2 to 100	24316	124	24440	0.51		0.937	0.130	-0.470	0.638	0.714	1.229	
unraced	774	5	779	0.64		1.012	0.484	0.030	0.980	0.396	2.585	
horse data incomplete	42213	268	42481	0.63		1.232	0.148	1.730	0.084	0.973	1.560	
					sigma	1.593	0.151			1.323	1.918	
					rho	0.435	0.047			0.347	0.528	0.000
**HORSE FELL PREVIOUSLY**												
no & unraced	65504	362	65866	0.55		**ref**						
yes	33369	206	33575	0.61		1.293	0.136	2.450	0.014	1.052	1.589	
horse data incomplete	42213	268	42481	0.63		1.239	0.119	2.240	0.025	1.027	1.496	
					sigma	1.634	0.150			1.365	1.955	
					rho	0.448	0.045			0.361	0.538	0.019
**HORSE STARTS IN LAST 365 DAYS**												
	median	IQR	range									
	5	4 to 7	0 to 30									
0 to 4	37946	239	38185	0.63		**ref**						0.119
5 to 7	39625	237	39862	0.59		1.010	0.099	0.100	0.922	0.833	1.223	
8 to 30	20528	87	20615	0.42		0.754	0.103	-2.070	0.039	0.577	0.985	
unraced	774	5	779	0.64		0.930	0.437	-0.150	0.878	0.371	2.334	
horse data incomplete	42213	268	42481	0.63		1.086	0.109	0.810	0.416	0.891	1.323	
					sigma	1.472	0.165			1.182	1.833	
					rho	0.397	0.054			0.298	0.505	0.000

**DAYS SINCE LAST RACE**												
	median	IQR	range									
	30	19 to 62	1 to 2,834									
1 to 365	94828	534	95362	0.56		**ref**						0.149
more than 365	3271	29	3300	0.88		1.434	0.288	1.790	0.073	0.967	2.125	
unraced	774	5	779	0.64		0.978	0.459	-0.050	0.961	0.390	2.452	
horse data incomplete	42213	268	42481	0.63		1.154	0.100	1.650	0.099	0.973	1.369	
					sigma	1.512	0.156			1.236	1.850	
					rho	0.410	0.050			0.317	0.510	0.000
**AGE AT START**												
	median	IQR	range									
	8	7 to 9	4 to 17									
4 to 7	57830	321	58151	0.55								
8	29102	176	29278	0.60								
9	22912	141	23053	0.61								
more than 9	31242	198	31440	0.63								
												0.000
per year						1.112	0.029	4.020	0.000	1.056	1.171	
					sigma	1.920	0.161			1.630	2.262	
					rho	0.529	0.042			0.447	0.609	0.000
**SEX AT START**												
male	129316	780	130096	0.60		**ref**						0.026
female	11770	56	11826	0.47		0.704	0.111	-2.230	0.026	0.518	0.958	
					sigma	1.574	0.150			1.306	1.898	
					rho	0.430	0.047			0.341	0.523	0.000
**WEIGHT CARRIED**												
	median	IQR	range									
	157	150 to 162	130 to 176									
130 to 162	109830	630	110460	0.57		**ref**						0.076
163 to 176	31256	206	31462	0.65		1.161	0.098	1.770	0.076	0.984	1.369	
					sigma	1.547	0.153			1.275	1.877	
					rho	0.421	0.048			0.331	0.517	0.000

**HORSE PENALTY**												
no	139745	833	140578	0.59		**ref**						0.090
yes	1341	3	1344	0.22		0.370	0.217	-1.700	0.090	0.117	1.166	
					sigma	1.538	0.153			1.267	1.869	
					rho	0.418	0.048			0.328	0.515	0.000
**CHANGE GOING**												
firmer surface	43964	269	44233	0.61		**ref**						0.025
same going	51254	326	51580	0.63		1.051	0.089	0.590	0.556	0.890	1.242	
softer surface	43300	217	43517	0.50		0.816	0.076	-2.180	0.029	0.680	0.980	
unraced	772	5	777	0.64		0.900	0.424	-0.220	0.824	0.358	2.267	
horse data incomplete	1796	19	1815	1.05		1.455	0.371	1.470	0.141	0.883	2.398	
					sigma	1.506	0.158			1.226	1.849	
					rho	0.408	0.051			0.313	0.510	0.000
**HORSE FELL RACE**												
no	135687	464	136151	0.34		**ref**						0.000
yes	5399	372	5771	6.45		21.171	1.851	34.910	0.000	17.837	25.128	
					sigma	0.882	0.360			0.396	1.962	
					rho	0.191	0.126			0.046	0.539	0.000
**HORSE FELL LAST**												
no	95192	541	95733	0.57		**ref**						0.337
yes	2907	22	2929	0.75		1.284	0.290	1.110	0.268	0.825	1.999	
unraced	774	5	779	0.64		0.962	0.452	-0.080	0.934	0.383	2.417	
horse data incomplete	42213	268	42481	0.63		1.145	0.100	1.560	0.119	0.966	1.359	
					sigma	1.536	0.153			1.264	1.868	
					rho	0.418	0.048			0.327	0.515	0.000
**HORSE PREV LTI**												
no	96102	544	96646	0.56		**ref**						0.074
yes	1997	19	2016	0.94		1.848	0.514	2.210	0.027	1.071	3.188	
unraced	774	5	779	0.64		0.966	0.454	-0.070	0.941	0.384	2.428	
unknown	42213	268	42481	0.63		1.154	0.101	1.640	0.101	0.972	1.369	
					sigma	1.545	0.152			1.274	1.874	
					rho	0.421	0.048			0.330	0.516	0.000

**DAYS SINCE LAST LTI**												
uninjured	96102	544	96646	0.56		**ref**						0.095
0 to 365	300	2	302	0.66		1.338	1.013	0.380	0.701	0.303	5.901	
366 to 730	620	4	624	0.64		1.147	0.624	0.250	0.801	0.395	3.332	
more than 730	1077	13	1090	1.19		2.498	0.847	2.700	0.007	1.286	4.856	
unraced	774	5	779	0.64		0.965	0.454	-0.080	0.939	0.384	2.426	
unknown	42213	268	42481	0.63		1.154	0.101	1.640	0.100	0.973	1.370	
					sigma	1.550	0.152			1.279	1.879	
					rho	0.422	0.048			0.332	0.518	0.000
**YEAR**												
2010	10474	68	10542	0.65		**ref**						0.229
2011	11399	82	11481	0.71		1.199	0.209	1.040	0.298	0.852	1.687	
2012	10615	75	10690	0.70		1.213	0.219	1.070	0.287	0.851	1.729	
2013	11304	56	11360	0.49		0.849	0.165	-0.840	0.401	0.580	1.243	
2014	10648	48	10696	0.45		0.772	0.156	-1.280	0.202	0.519	1.148	
2015	10739	57	10796	0.53		0.920	0.179	-0.430	0.671	0.628	1.349	
2016	10874	59	10933	0.54		0.957	0.186	-0.230	0.821	0.654	1.400	
2017	10820	57	10877	0.52		0.928	0.182	-0.380	0.705	0.633	1.363	
2018	10710	64	10774	0.59		1.046	0.199	0.240	0.814	0.720	1.519	
2019	10361	68	10429	0.65		1.142	0.215	0.710	0.480	0.790	1.650	
2020	8154	43	8197	0.52		0.897	0.188	-0.520	0.605	0.595	1.353	
2021	10722	79	10801	0.73		1.287	0.235	1.380	0.168	0.899	1.841	
2022	9913	51	9964	0.51		0.900	0.182	-0.520	0.602	0.606	1.337	
2023	4353	29	4382	0.66		1.173	0.281	0.660	0.507	0.733	1.877	
					sigma	1.506	0.161			1.221	1.857	
					rho	0.408	0.052			0.312	0.512	0.000

**RACE MONTH**												
January	13187	64	13251	0.48		**ref**						0.078
February	13786	73	13859	0.53		1.106	0.194	0.570	0.568	0.783	1.560	
March	18425	121	18546	0.65		1.382	0.220	2.040	0.042	1.012	1.888	
April	15838	103	15941	0.65		1.392	0.229	2.010	0.044	1.009	1.921	
May	12729	82	12811	0.64		1.382	0.239	1.870	0.062	0.984	1.940	
June	7016	53	7069	0.75		1.627	0.315	2.510	0.012	1.113	2.378	
July	6034	38	6072	0.63		1.352	0.288	1.410	0.157	0.890	2.054	
August	4739	36	4775	0.75		1.657	0.361	2.320	0.021	1.081	2.539	
September	4652	27	4679	0.58		1.237	0.294	0.890	0.372	0.776	1.972	
October	11700	73	11773	0.62		1.301	0.230	1.490	0.137	0.920	1.840	
November	17147	82	17229	0.48		0.990	0.169	-0.060	0.952	0.708	1.384	
December	15833	84	15917	0.53		1.099	0.187	0.550	0.581	0.787	1.534	
					sigma	1.568	0.151			1.299	1.893	
					rho	0.428	0.047			0.339	0.521	0.000
**SEASON**												
winter	42806	221	43027	0.51		**ref**						0.003
spring	46992	306	47298	0.65		1.293	0.119	2.800	0.005	1.080	1.548	
summer	17789	127	17916	0.71		1.435	0.170	3.050	0.002	1.138	1.810	
autumn	33499	182	33681	0.54		1.056	0.109	0.520	0.602	0.862	1.293	
					sigma	1.566	0.151			1.297	1.892	
					rho	0.427	0.047			0.338	0.521	0.000
**JUMP SEASON**												
core jump season	110568	627	111195	0.56		**ref**						0.012
summer jump season	30518	209	30727	0.68		1.237	0.105	2.500	0.012	1.047	1.460	
					sigma	1.547	0.152			1.277	1.875	
					rho	0.421	0.048			0.331	0.517	0.000
**RACE TIME**												
morning	25532	133	25665	0.52		**ref**						0.038
afternoon	73499	428	73927	0.58		1.159	0.119	1.430	0.152	0.947	1.418	
evening	42055	275	42330	0.65		1.320	0.146	2.510	0.012	1.063	1.640	
					sigma	1.570	0.151			1.300	1.895	
					rho	0.428	0.047			0.339	0.522	0.000

FIELD SIZE

	median	IQR	range									
	8	6 to 10	1 to 40									
1 to 5	24079	120	24199	0.50								
6 to 10	81768	476	82244	0.58								
11 to 15	26897	164	27061	0.61								
16 to 20	5463	39	5502	0.71								
21 to 25	1677	16	1693	0.95								
26 to 30	696	12	708	1.69								
35 to 40	506	9	515	1.75								
												0.000
per runner						1.037	0.007	5.060	0.000	1.022	1.052	
					sigma	1.597	0.150	1.329	1.919			
					rho	0.437	0.046	0.349	0.528			

PLACES PAID

3 to 5	112567	656	113223	0.58		**ref**						0.222
6 to 10	28519	180	28699	0.63		1.118	0.103	1.220	0.222	0.934	1.339	
					sigma	1.553	0.152			1.282	1.883	
					rho	0.423	0.048			0.333	0.519	0.000

RACE DISTANCE (100m)

	median	IQR	range									
	46.6	42.8 to 53.1	33.3 to 79.2									
33.3 to 42.8	35482	179	35661	0.50		**ref**						0.005
42.9 to 46.6	35423	246	35669	0.69		1.408	0.147	3.270	0.001	1.147	1.729	
46.7 to 53.1	35024	193	35217	0.55		1.142	0.128	1.180	0.237	0.917	1.423	
53.2 to 79.2	35157	218	35375	0.62		1.327	0.149	2.530	0.012	1.065	1.654	
					sigma	1.569	0.153			1.296	1.900	
					rho	0.428	0.048			0.338	0.523	

**GOING**												
firm and good to firm	7775	55	7830	0.70		1.161	0.186	0.930	0.351	0.849	1.588	
good	53461	366	53827	0.68		1.164	0.106	1.660	0.097	0.973	1.393	
good to soft	33358	200	33558	0.60		**ref**						0.000
soft	32474	163	32637	0.50		0.828	0.090	-1.730	0.084	0.669	1.026	
heavy	14018	52	14070	0.37		0.614	0.098	-3.050	0.002	0.448	0.840	
					sigma	1.545	0.153			1.272	1.877	
					rho	0.421	0.048			0.330	0.517	0.000
**TRACK DIRECTION**												
figure 8 track	4929	20	4949	0.40		**ref**						0.013
left-handed	89467	566	90033	0.63		1.583	0.373	1.950	0.051	0.998	2.513	
right-handed	46690	250	46940	0.53		1.306	0.314	1.110	0.267	0.815	2.092	
					sigma	1.554	0.152			1.284	1.882	
					rho	0.423	0.048			0.334	0.518	0.000
**RACE CLASS**												
1	12373	93	12466	0.75		1.520	0.212	2.990	0.003	1.156	1.999	
2	12685	80	12765	0.63		1.289	0.184	1.780	0.076	0.974	1.704	
3	32930	172	33102	0.52		**ref**						0.029
4	50368	295	50663	0.58		1.102	0.112	0.960	0.339	0.903	1.345	
5	28041	174	28215	0.62		1.234	0.144	1.800	0.072	0.981	1.552	
6	4689	22	4711	0.47		0.851	0.203	-0.680	0.499	0.533	1.358	
					sigma	1.597	0.151			1.328	1.922	
					rho	0.437	0.046			0.349	0.529	0.000
**RACE VALUE**												
	median	IQR	range									
	8700	6,000 to 15,000	1,150 to 1,000,000									
1,150 to 6,000	41426	230	41656	0.55		**ref**						0.090
6,001 to 8,700	29360	194	29554	0.66		1.206	0.126	1.800	0.072	0.983	1.480	
8,701 to 15,000	37048	202	37250	0.54		0.997	0.103	-0.030	0.978	0.815	1.221	
15,000 to 1,000,000	33252	210	33462	0.63		1.204	0.128	1.750	0.080	0.978	1.483	
					sigma	1.564	0.152			1.292	1.892	
					rho	0.426	0.048			0.337	0.521	0.000

**PATTERN RACE**												
no	131707	764	132471	0.58		**ref**						0.019
yes	9379	72	9451	0.76		1.373	0.185	2.350	0.019	1.054	1.789	
					sigma	1.551	0.152			1.279	1.880	
					rho	0.422	0.048			0.332	0.518	0.000
**CLAIMING**												
no	141060	835	141895	0.59		**ref**						0.090
yes	26	1	27	3.70		6.993	8.019	1.700	0.090	0.739	66.174	
					sigma	1.536	0.153			1.264	1.867	
					rho	0.418	0.048			0.327	0.514	0.000
**WINNING SPEED**												
	median	IQR	range									
	14.1	13.7 to 14.5	4.2 to 31.7									
≤14.5m/s	99678	563	100241	0.56		**ref**						0.044
>14.5m/s	41408	273	41681	0.65		1.169	0.091	2.010	0.044	1.004	1.362	
					sigma	1.532	0.153			1.260	1.863	
					rho	0.416	0.049			0.325	0.513	0.000
**COURSE RUNNERS 365 DAYS**												
	median	IQR	range									
	1120	871 to 1,561	0 to 7,269									
0 to 871	35364	234	35598	0.66		**ref**						0.130
872 to 1,120	35259	188	35447	0.53		0.790	0.080	-2.320	0.021	0.647	0.964	
1,121 to 1,561	35205	212	35417	0.60		0.892	0.088	-1.150	0.249	0.735	1.083	
1,562 to 7,269	35258	202	35460	0.57		0.859	0.086	-1.520	0.128	0.706	1.045	
					sigma	1.545	0.152			1.274	1.874	
					rho	0.421	0.048			0.330	0.516	0.000
**TRAINER COUNTRY**												
Great Britain	137300	785	138085	0.57		**ref**						0.000
Other	3786	51	3837	1.33		2.328	0.376	5.240	0.000	1.697	3.195	
					sigma	1.445	0.159			1.164	1.794	
					rho	0.388	0.052			0.292	0.495	0.000

TRAINER YEARS RACED

	median	IQR	range									
	18	11 to 24	0 to 36									
0 to 11	37612	205	37817	0.54		**ref**						0.000
12 to 18	35363	191	35554	0.54		1.001	0.111	0.010	0.992	0.806	1.243	
19 to 24	32375	196	32571	0.60		1.134	0.126	1.130	0.257	0.912	1.410	
25 to 26	31675	193	31868	0.61		1.172	0.133	1.410	0.159	0.939	1.463	
trainer data incomplete	4061	51	4112			2.332	0.407	4.850	0.000	1.656	3.284	
					sigma	1.475	0.157			1.197	1.818	
					rho	0.398	0.051			0.304	0.501	0.000

TRAINER START NO

	median	IQR	range									
	2298	662 to 6,205	1 to 25,424									
1 to 662	34291	177	34468	0.51		**ref**						0.000
663 to 2,298	34276	178	34454	0.52		1.001	0.116	0.010	0.996	0.797	1.257	
2,299 to 6,205	34221	224	34445	0.65		1.304	0.146	2.370	0.018	1.047	1.623	
6,206 to 25,424	34237	206	34443	0.60		1.161	0.132	1.310	0.189	0.929	1.452	
trainer details unreliable	4061	51	4112	1.24		2.430	0.429	5.030	0.000	1.719	3.434	
					sigma	1.455	0.158			1.176	1.801	
					rho	0.392	0.052			0.296	0.497	0.000

TRAINER PLACE RATE

	median	IQR	range									
	33.5	29.5 to 36.6	0 to 100									
0 to 36.6	102307	614	102921	0.60		**ref**						0.000
36.7 to 100	34057	168	34225	0.49		0.780	0.076	-2.550	0.011	0.644	0.944	
trainer first start	661	3	664	0.45		0.700	0.416	-0.600	0.548	0.218	2.245	
trainer details unreliable	4061	51	4112	1.24		2.052	0.336	4.390	0.000	1.489	2.828	
					sigma	1.486	0.155			1.210	1.824	
					rho	0.402	0.050			0.308	0.503	0.000

**JOCKEY LICENSE TYPE**												
Conditional or amateur	43016	243	43259	0.56		**ref**						0.006
Jump	94074	551	94625	0.58		1.011	0.083	0.130	0.896	0.860	1.188	
Unknown	3996	42	4038	1.04		1.758	0.321	3.090	0.002	1.229	2.513	
					sigma	1.492	0.160			1.209	1.840	
					rho	0.403	0.052			0.308	0.507	0.000
**JOCKEY YEARS RACED**												
	median	IQR	range									
	9	6 to 14	0 to 32									
0 to 6	42926	234	43160	0.54		**ref**						0.206
7 to 9	29672	175	29847	0.59		1.079	0.114	0.720	0.475	0.877	1.327	
10 to 14	36702	243	36945	0.66		1.222	0.120	2.050	0.040	1.009	1.481	
15 to 32	31786	184	31970	0.58		1.048	0.110	0.450	0.655	0.853	1.289	
					sigma	1.539	0.153			1.266	1.870	
					rho	0.418	0.048			0.328	0.515	0.000
