# Supplementary material for: Risk factors for fatality in jump racing Thoroughbreds in Great Britain (2010–2023)
Source: Equine Vet J. 2024 Dec 12;57(4):870–7. doi: 10.1111/evj.14450 (PMC12135743; doi:10.1111/evj.14450)
Supplement: Supplementary file 3 — Table S3: Descriptive statistics and results of univariable logistic regression for fatality in hurdle racing. [file EVJ-57-870-s001.pdf]

Equine Veterinary Journal BEVA

Table S3: Descriptive statistics and results of univariable logistic regression for fatality in hurdle racing.

	DESCRIPTIVE STATISTICS					UNIVARIABLE (RANDOM EFFECT - HORSE)						
	non-fatal	fatal	total	% fatal		OR	SE	Z	P	95% CI		LRT
**MAKING FIRST START**												
yes	183248	805	184053	0.44		**ref**						0.135
no	4875	28	4903	0.57		1.287	0.251	1.290	0.197	0.877	1.886	
horse data incomplete	53267	263	53530	0.49		1.126	0.082	1.630	0.102	0.977	1.299	
					sigma_u	0.755	0.497			0.208	2.741	
					rho	0.148	0.166			0.013	0.695	0.259
**YEARS IN RACING**												
	median	IQR	range									
	2	1 to 3	0 to 12									
0 to 1	82927	344	83271	0.41		**ref**						0.002
2	40621	177	40798	0.43		1.100	0.108	0.980	0.328	0.908	1.333	
3	25115	101	25216	0.40		1.050	0.129	0.400	0.690	0.826	1.335	
4 to 12	39460	211	39671	0.53		1.507	0.168	3.680	0.000	1.212	1.875	
horse data incomplete	53267	263	53530	0.49		1.261	0.115	2.540	0.011	1.054	1.509	
					sigma_u	1.382	0.219			1.013	1.885	
					rho	0.367	0.074			0.238	0.519	0.005
**HORSE STARTS IN LAST 30 DAYS**												
	median	IQR	range									
	1	0 to 1	0 to 5									
0	89687	425	90112	0.47		**ref**						0.035
1 to 5	93561	380	93941	0.40		0.859	0.061	-2.150	0.032	0.747	0.987	
making first start	4875	28	4903	0.57		1.204	0.236	0.950	0.343	0.820	1.769	
horse data incomplete	53267	263	53530	0.49		1.044	0.083	0.540	0.587	0.894	1.220	
					sigma_u	0.533	0.276			0.193	1.471	
					rho	0.080	0.076			0.011	0.397	0.341

**HORSE STARTS IN LAST 60 DAYS**												
	median	IQR	range									
	1	0 to 2	0 to 8									
0	46005	230	46235	0.50		**ref**						0.030
1 to 8	137243	575	137818	0.42		0.840	0.066	-2.230	0.026	0.720	0.979	
making first start	4875	28	4903	0.57		1.143	0.230	0.660	0.508	0.770	1.695	
horse data incomplete	53267	263	53530	0.49		0.990	0.090	-0.110	0.914	0.828	1.184	
					sigma_u	0.527	0.276			0.189	1.470	
					rho	0.078	0.075			0.011	0.396	0.345
**HORSE STARTS IN LAST 365 DAYS**												
	median	IQR	range									
	5	3 to 7	0 to 37									
0 to 3	5411	38	5449	0.70		**ref**						0.015
4 to 5	61014	274	61288	0.45		0.640	0.111	-2.570	0.010	0.455	0.899	
6 to 7	64061	282	64343	0.44		0.627	0.109	-2.690	0.007	0.447	0.881	
8 to 37	52762	211	52973	0.40		0.570	0.101	-3.170	0.002	0.403	0.807	
making first start	4875	28	4903	0.57		0.818	0.204	-0.810	0.420	0.501	1.334	
horse data incomplete	53267	263	53530	0.49		0.704	0.123	-2.020	0.044	0.500	0.990	
					sigma_u	0.233	0.250			0.028	1.920	
					rho	0.016	0.034			0.000	0.528	0.454
**DAYS SINCE LAST RACE**												
	median	IQR	range									
	30	19 to 61	1 to 2,632									
1 to 365	177837	767	178604	0.43		**ref**						0.007
more than 365	5411	38	5449	0.70		1.621	0.271	2.890	0.004	1.168	2.250	
making first start	4875	28	4903	0.57		1.324	0.256	1.450	0.147	0.906	1.934	
horse data incomplete	53267	263	53530	0.49		1.146	0.083	1.880	0.060	0.994	1.320	
					sigma_u	0.490	0.304			0.145	1.654	
					rho	0.068	0.079			0.006	0.454	0.368

**AGE AT START**												
	median	IQR	range									
	6	5 to 7	3 to 18									
3 to 5	92067	342	92409	0.37								
6	58530	286	58816	0.49								
7	38945	197	39142	0.50								
8 to 18	51848	271	52119	0.52								
												0.000
per year						1.138	0.025	5.860	0.000	1.090	1.188	
					sigma_u	1.710	0.166			1.413	2.069	
					rho	0.470	0.048			0.378	0.565	0.000
**SEX AT START**												
male	188132	879	189011	0.47		**ref**						0.071
female	53258	217	53475	0.41		0.871	0.067	-1.810	0.071	0.750	1.012	
					sigma_u	0.481	0.153			0.257	0.899	
					rho	0.066	0.039			0.020	0.197	0.286
**BHA RATING**												
	median	IQR	range									
	106	96 to 119	1o to 175									
												0.037
1 to 96	49926	256	50182	0.51		1.113	0.118	1.010	0.311	0.905	1.370	
76 to 106	33458	156	33614	0.46		**ref**						
107 to 119	48369	198	48567	0.41		0.862	0.096	-1.340	0.179	0.693	1.071	
120 to 175	37052	175	37227	0.47		0.998	0.116	-0.020	0.985	0.795	1.253	
unrated	72585	311	72896	0.43		0.827	0.095	-1.650	0.099	0.660	1.036	
					sigma_u	1.338	0.269			0.902	1.982	
					rho	0.352	0.092			0.198	0.544	0.021
**TOP BAND HANDICAP**												
no	171727	798	172525	0.46		**ref**						0.076
yes	69663	298	69961	0.43		0.867	0.070	-1.780	0.076	0.740	1.015	
					sigma_u	1.166	0.302			0.702	1.937	
					rho	0.292	0.107			0.130	0.533	0.059
**UNRATED**												
no	168805	785	169590	0.46		**ref**						0.049
yes	72585	311	72896	0.43		0.843	0.073	-1.970	0.049	0.711	0.999	
					sigma_u	1.278	0.288			0.822	1.987	
					rho	0.332	0.100			0.170	0.545	0.035

**HORSE PENALTY**												
no	239742	1093	240835	0.45		**ref**						0.118
yes	1648	3	1651	0.18		0.404	0.234	-1.560	0.118	0.130	1.260	
					sigma_u	0.791	0.468			0.248	2.523	
					rho	0.160	0.159			0.018	0.659	0.236
**RACING OUT OF HANDICAP**												
no	131551	593	132144	0.45		**ref**						0.372
yes	9327	51	9378	0.54		1.214	0.178	1.320	0.186	0.911	1.620	
weight for age	100512	452	100964	0.45		0.986	0.062	-0.230	0.820	0.871	1.116	
					sigma_u	0.592	0.142			0.369	0.948	
					rho	0.096	0.042			0.040	0.214	0.224
**EYE COVER**												
no	205583	952	206535	0.46		**ref**						0.139
yes	35807	144	35951	0.40		0.874	0.079	-1.480	0.139	0.732	1.045	
					sigma_u	0.513	0.383			0.119	2.216	
					rho	0.074	0.102			0.004	0.599	0.380
**CHANGE WEIGHT CAT**												
more weight	89,084	394	89,478	0.44		**ref**						0.098
same weight	19,712	70	19,782	0.35		0.801	0.104	-1.710	0.088	0.620	1.033	
less weight	74,452	341	74,793	0.46		1.037	0.077	0.490	0.628	0.896	1.199	
making first start	4,875	28	4,903	0.57		1.287	0.253	1.280	0.199	0.875	1.892	
horse data incomplete	53,267	263	53,530	0.49		1.118	0.090	1.380	0.167	0.954	1.309	
					sigma_u	0.586	0.173			0.329	1.044	
					rho	0.094	0.050			0.032	0.249	0.256
**CHANGE DISTANCE**												
longer race	89,084	394	89,478	0.44		**ref**						0.098
same distance	19,712	70	19,782	0.35		0.801	0.104	-1.710	0.088	0.620	1.033	
shorter race	74,452	341	74,793	0.46		1.037	0.077	0.490	0.628	0.896	1.199	
making first start	4,875	28	4,903	0.57		1.287	0.253	1.280	0.199	0.875	1.892	
horse data incomplete	53,267	263	53,530	0.49		1.118	0.090	1.380	0.167	0.954	1.309	
					sigma_u	0.586	0.173			0.329	1.044	
					rho	0.094	0.050			0.032	0.249	0.256

**CHANGE GOING**												
firmer going	57,572	296	57,868	0.51		**ref**						0.001
same going	66,856	294	67,150	0.44		0.856	0.071	-1.880	0.060	0.728	1.007	
softer going	58,820	215	59,035	0.36		0.710	0.064	-3.800	0.000	0.595	0.847	
making first start	4,875	28	4,903	0.57		1.099	0.221	0.470	0.638	0.742	1.629	
horse data incomplete	53,267	263	53,530	0.49		0.962	0.083	-0.440	0.657	0.812	1.140	
					sigma_u	0.760	0.494			0.213	2.714	
					rho	0.149	0.165			0.014	0.691	0.255
**HORSE FELL RACE**												
no	237091	734	237825	0.31		**ref**						0.000
yes	4299	362	4661	7.77		29.582	3.341	29.990	0.000	23.709	36.911	
					sigma_u	0.930	0.425			0.380	2.277	
					rho	0.208	0.151			0.042	0.612	0.165
**HORSE PREVIOUS LTI**												
no	181283	790	182073	0.43		**ref**						0.033
yes	1965	15	1980	0.76		1.776	0.470	2.170	0.030	1.058	2.983	
making first start	4875	28	4903	0.57		1.310	0.253	1.400	0.162	0.897	1.914	
horse data incomplete	53267	263	53530	0.49		1.134	0.082	1.750	0.080	0.985	1.306	
					sigma_u	0.484	0.148			0.266	0.880	
					rho	0.066	0.038			0.021	0.191	0.276
**DAYS SINCE LAST LTI**												
no injury	181283	790	182073	0.43		**ref**						0.088
0 to 365 days	390	2	392	0.51		1.191	0.848	0.240	0.806	0.295	4.809	
366 to 730 days	684	5	689	0.73		1.701	0.770	1.170	0.241	0.700	4.131	
more than 730 days	891	8	899	0.89		2.094	0.755	2.050	0.040	1.033	4.245	
making first start	4875	28	4903	0.57		1.310	0.253	1.400	0.162	0.897	1.914	
horse data incomplete	53267	263	53530	0.49		1.134	0.082	1.750	0.080	0.985	1.306	
					sigma_u	0.484	0.147			0.267	0.878	
					rho	0.067	0.038			0.021	0.190	0.275

**MONTH**												
January	23895	76	23971	0.32		**ref**						0.000
February	23053	84	23137	0.36		1.145	0.182	0.850	0.394	0.838	1.565	
March	29987	174	30161	0.58		1.834	0.254	4.370	0.000	1.397	2.406	
April	23234	119	23353	0.51		1.627	0.241	3.280	0.001	1.217	2.176	
May	19298	113	19411	0.58		1.874	0.282	4.180	0.000	1.396	2.516	
June	12255	68	12323	0.55		1.774	0.300	3.390	0.001	1.273	2.471	
July	11706	60	11766	0.51		1.640	0.287	2.830	0.005	1.163	2.310	
August	9545	55	9600	0.57		1.845	0.331	3.410	0.001	1.297	2.623	
September	9812	48	9860	0.49		1.572	0.294	2.420	0.015	1.090	2.267	
October	21926	106	22032	0.48		1.536	0.233	2.830	0.005	1.141	2.068	
November	30170	105	30275	0.35		1.099	0.167	0.620	0.535	0.816	1.479	
December	26509	88	26597	0.33		1.047	0.165	0.290	0.769	0.769	1.426	
					sigma_u	1.016	0.283			0.588	1.754	
					rho	0.239	0.101			0.095	0.483	0.098
**METEROLOGICAL SEASON**												
winter	73457	248	73705	0.34		**ref**						0.000
spring	72519	406	72925	0.56		1.671	0.136	6.290	0.000	1.424	1.961	
summer	33506	183	33689	0.54		1.640	0.163	4.960	0.000	1.349	1.993	
autumn	61908	259	62167	0.42		1.248	0.112	2.460	0.014	1.046	1.488	
					sigma_u	0.982	0.345			0.493	1.954	
					rho	0.227	0.123			0.069	0.537	0.121
**JUMP SEASON**												
core jump season	188586	800	189386	0.42		**ref**						0.000
summer jump season	52804	296	53100	0.56		1.333	0.093	4.130	0.000	1.163	1.528	
					sigma_u	0.946	0.358			0.451	1.985	
					rho	0.214	0.127			0.058	0.545	0.138
**RACE TIME**												
morning	65662	269	65931	0.41		**ref**						0.000
afternoon	108958	459	109417	0.42		1.042	0.081	0.530	0.599	0.894	1.215	
late afternoon/evening	66770	368	67138	0.55		1.378	0.115	3.850	0.000	1.171	1.623	
					sigma_u	1.064	0.304			0.608	1.861	
					rho	0.256	0.109			0.101	0.513	0.077

**GOING**												
firm and good to firm	14193	94	14287	0.66		1.495	0.183	3.290	0.001	1.177	1.900	
good	92650	517	93167	0.55		1.260	0.098	2.980	0.003	1.082	1.467	
good to soft	56364	251	56615	0.44		**ref**						0.000
soft	55460	182	55642	0.33		0.735	0.072	-3.150	0.002	0.607	0.890	
heavy	22723	52	22775	0.23		0.513	0.078	-4.370	0.000	0.380	0.692	
					sigma_u	0.782	0.139			0.553	1.107	
					rho	0.157	0.047			0.085	0.271	0.121
**TRACK DIRECTION**												
figure 8	8330	30	8360	0.36		**ref**						0.045
left-handed	150126	720	150846	0.48		1.330	0.250	1.520	0.129	0.921	1.921	
right-handed	82934	346	83280	0.42		1.156	0.221	0.750	0.451	0.794	1.682	
					sigma_u	0.823	0.441			0.288	2.351	
					rho	0.171	0.152			0.025	0.627	0.215
**RACE VALUE**												
	median	IQR	range									
	6100	5000 to 9000	2000 to 470250									
2000 to 5000	84,907	395	85,302	0.46		**ref**						0.106
5001 to 6100	35,807	188	35,995	0.52		1.132	0.101	1.380	0.167	0.950	1.349	
6101 to 9000	64,455	273	64,728	0.42		0.914	0.073	-1.130	0.260	0.782	1.069	
9001 to 470250	56,221	240	56,461	0.43		0.923	0.077	-0.960	0.335	0.783	1.087	
					sigma_u	0.724	0.532			0.172	3.054	
					rho	0.138	0.174			0.009	0.739	0.275
**HURDLE TYPE**												
birch	182701	815	183516	0.44		**ref**						0.004
brush	16442	102	16544	0.62		1.393	0.147	3.140	0.002	1.133	1.714	
padded	42247	179	42426	0.42		0.952	0.079	-0.600	0.552	0.809	1.120	
					sigma_u	0.590	0.171			0.334	1.042	
					rho	0.096	0.050			0.033	0.248	0.254
**CONDITIONAL RACE**												
no	225468	1007	226475	0.44		**ref**						0.035
yes	15922	89	16011	0.56		1.268	0.143	2.110	0.035	1.017	1.581	
					sigma_u	0.910	0.378			0.403	2.055	
					rho	0.201	0.134			0.047	0.562	0.158

**MAIDEN RACE**													
	no	211723	937	212660	0.44		**ref**						0.036
	yes	29667	159	29826	0.53		1.205	0.107	2.100	0.036	1.012	1.434	
						sigma_u	0.457	0.918			0.009	23.420	
						rho	0.060	0.226			0.000	0.994	0.417
**NOVICE RACE**													
	no	168692	799	169491	0.47		**ref**						0.010
	yes	72698	297	72995	0.41		0.827	0.061	-2.580	0.010	0.716	0.956	
						sigma_u	1.141	0.279			0.707	1.841	
						rho	0.283	0.099			0.132	0.508	
**SELLER**													
	no	235703	1058	236761	0.45		**ref**						0.015
	yes	5687	38	5725	0.66		1.508	0.253	2.440	0.015	1.084	2.096	
						sigma_u	0.876	0.399			0.359	2.139	
						rho	0.189	0.140			0.038	0.582	0.178
**CLAIMING**													
	no	240289	1087	241376	0.45		**ref**						0.079
	yes	1101	9	1110	0.81		1.814	0.615	1.760	0.079	0.933	3.527	
						sigma_u	0.830	0.435			0.297	2.320	
						rho	0.173	0.150			0.026	0.621	0.209
**RESTRICTED FILLIES**													
	no	218058	1010	219068	0.46		**ref**						0.040
	yes	23332	86	23418	0.37		0.791	0.090	-2.050	0.040	0.633	0.990	
						sigma_u	0.853	0.415			0.329	2.214	
						rho	0.181	0.144			0.032	0.598	0.193
**WINNING SPEED**													
		median	IQR	range									
		14,5	14.1-15.1	10.1-40.5									
	≤14.5m/s	103,210	362	103,572	0.35		**ref**						0.000
	>14.5m/s	138,180	734	138,914	0.53		1.519	0.099	6.440	0.000	1.337	1.725	
						sigma_u	0.827	0.444			0.288	2.368	
						rho	0.172	0.153			0.025	0.630	0.213

**COURSE SPEED**												
Galloping	109939	456	110395	0.41		**ref**						0.009
Stiff, tight, sharp, very sharp	131451	640	132091	0.48		1.175	0.072	2.620	0.009	1.041	1.326	
					sigma_u	0.485	0.145			0.270	0.869	
					rho	0.067	0.037			0.022	0.187	0.272
**COURSE RUNNERS 180 DAYS**												
	median	IQR	range									
	569	369 to 814	0 to 4579									
0 to 369	60,458	246	60,704	0.41		**ref**						0.033
370 to 569	60,354	264	60,618	0.44		1.075	0.096	0.810	0.420	0.902	1.280	
570 to 814	60,285	313	60,598	0.52		1.275	0.109	2.830	0.005	1.078	1.508	
815 to 4579	60,293	273	60,566	0.45		1.111	0.098	1.190	0.236	0.934	1.321	
					sigma_u	0.795	0.469			0.250	2.525	
					rho	0.161	0.159			0.019	0.660	0.234
**TRAINER LICENSE**												
Jump or Dual	226,485	1,016	227,501	0.45		**ref**						0.117
Permit	9,317	44	9,361	0.47		1.057	0.167	0.350	0.723	0.776	1.440	
Unknown	5,588	36	5,624	0.64		1.424	0.246	2.050	0.040	1.016	1.997	
					sigma_u	0.746	0.505			0.198	2.813	
					rho	0.145	0.168			0.012	0.706	0.265
**TRAINER COUNTRY**												
Great Britain	235817	1060	236877	0.45		**ref**						0.040
Other	5573	36	5609	0.64		1.425	0.246	2.060	0.040	1.017	1.998	
					sigma_u	0.737	0.515			0.187	2.902	
					rho	0.142	0.170			0.011	0.719	0.269
**TRAINER WIN RATE**												
	median	IQR	range									
	11	7.8 to 14.6	0 to 100									
0 to 14.6	181078	787	181865	0.43	inc. first start	**ref**						0.017
14.7 to 100	54000	269	54269	0.50		1.145	0.082	1.900	0.057	0.996	1.317	
trainer first start	52	0	52	0.00								
trainer data incomplete	6260	40	6300	0.63		1.463	0.239	2.330	0.020	1.062	2.016	
					sigma_u	0.512	0.281			0.174	1.504	
					rho	0.074	0.075			0.009	0.407	0.354

**JOCKEY LICENSE TYPE**												
Conditional and amateur	79057	397	79454	0.50								0.031
Jump	157348	675	158023	0.43		0.842	0.055	-2.620	0.009	0.741	0.958	
Unknown	4985	24	5009	0.48		0.930	0.200	-0.340	0.737	0.610	1.418	
					sigma_u	0.967	0.340			0.485	1.925	
					rho	0.221	0.121			0.067	0.530	0.120
**JOCKEY PLACE RATE**												
	median	IQR	range									
	29	20.2 to 33.8	0 to 100									
0 to 33.8	180741	844	181585	0.46								0.167
33.9 to 100	60264	249	60513	0.41		0.887	0.065	-1.640	0.100	0.769	1.023	
jockey first start	385	3	388	0.77		1.682	0.983	0.890	0.373	0.535	5.286	
					sigma_u	0.769	0.412			0.269	2.197	
					rho	0.152	0.138			0.022	0.595	0.244
